# Human Infection with Lymphocytic Choriomeningitis Virus

**DOI:** 10.3201/eid1606.100250

**Published:** 2010-06

**Authors:** Leslie L. Barton

**Affiliations:** University of Arizona, Tucson, Arizona, USA

**Keywords:** Lymphocytic choriomeningitis virus, zoonoses, human pathogen, viruses, letter

**To the Editor:** I read with great interest the article regarding lymphocytic choriomeningitis virus (LCMV) meningitis in a New York City resident (*1*). The authors’ conclusion that there is a need to ascertain the true incidence of LCMV infection is worthy of underscoring. Nearly 15 years ago, in this same journal, we described congenital LCMV as an unrecognized teratogen and recommended further “research to define the frequency of LCMV” (*2*). Five years later, we reiterated that recommendation when reporting acquired LCMV meningoencephalitis in an adolescent from Tucson, Arizona (*3*). Despite, or because of, the lack of prospective studies, the fact that this author has accrued data regarding >60 congenitally infected infants from all geographic areas in the United States during the past 15 years reinforces the concept that LCMV is a neglected pathogen whose time for more extensive study has indeed come.
